# Impact of low-dose prednisolone on bone synthesis and resorption in early rheumatoid arthritis: experiences from a two-year randomized study

**DOI:** 10.1186/ar2542

**Published:** 2008-11-05

**Authors:** Inga-Lill Engvall, Björn Svensson, Birgitta Tengstrand, Kerstin Brismar, Ingiäld Hafström, the BARFOT study group

**Affiliations:** 1 Department of Rheumatology, Karolinska Institutet at Karolinska University Hospital Huddinge, Hälsovägen 141, Stockholm, 141 86, Sweden; 2 Department of Rheumatology, University Hospital, Kioskgatan 3-9, Lund, 221 85, Sweden; 3 Department of Molecular Medicine and Surgery Rolf Luft Research Center for Diabetes and Endocrinology, Karolinska Institutet at Karolinska University Hospital, Solna, L1, Stockholm, 171 76, Sweden

## Abstract

**Introduction:**

Patients with rheumatoid arthritis (RA) have an increased frequency of osteoporosis, mainly because of increased bone resorption. Reduction of disease activity is suggested to reduce bone remodelling. It might also be possible that prednisolone treatment could cause this effect because prednisolone has been shown to arrest the development of joint destruction in early RA. Therefore, we examined the effects of low-dose prednisolone on serum concentrations of bone remodelling markers and insulin-like growth factor-1 (IGF-1) in RA patients in relation to bone mineral density.

**Methods:**

One hundred and fifty patients, 67% women, with early RA, mean disease duration of six months (95% confidence interval (CI) = three to eight months), who had participated in the BARFOT (Better Anti-Rheumatic FarmacOTherapy) low-dose prednisolone study were included. They had been randomised to either the P-group, who were treated with 7.5 mg prednisolone daily (n = 70, mean age = 51 years, 95% CI 48 to 54 years), or the NoP-group, who received no prednisolone (n = 80, mean age 58 years, 95% CI 56 to 61 years), when they started their first disease-modifying anti-rheumatic drug (DMARD). Serum samples were analysed at baseline, 3 and 12 months for procollagen type I N-terminal propeptide (P1NP), a marker of bone formation, and the C-telopeptide crosslaps of type I collagen (CTX-1) and C-terminal telopeptide of type I collagen (1CTP), markers of bone degradation. IGF-1 was analysed at baseline and after 12 months. Bone mineral density at the lumbar spine and femoral neck was assessed by dual-energy X-ray absorptiometry at baseline and after 24 months.

**Results:**

Levels of P1NP decreased rapidly in the P-group (p < 0.001). Levels of CTX-1 and 1CTP decreased in both treatment groups, but significantly more in the P-group (differences between groups p < 0.019 and p < 0.001, respectively). IGF-1 increased in the P-group (p < 0.001) but remained stable in the NoP-group. Bone mineral density decreased in the spine in both groups, significantly more in postmenopausal women from the P-group. Femur bone mineral density only decreased in the NoP-group.

**Conclusions:**

Low-dose prednisolone in early RA counteracts the negative impact of rheumatoid inflammation on bone tissue in the hip, a juxta-articular localisation. Thus bone mineral density was preserved in the femur in the P-group and 1CTP decreased rapidly. However, the systemic inflammatory consequences on bone could not be prevented in the lumbar spine, especially not in postmenopausal women, probably because of the combined effect of suppression of bone synthesis by prednisolone and the postmenopausal status.

## Introduction

Rheumatoid arthritis (RA) is associated with an increased risk of osteoporosis followed by an increased fracture rate [1-3]. This has been attributed to inflammatory activity and decreased physical activity but also to treatment with glucocorticoids and muscle weakness, which increases the likelihood of falls [1].

Bone is continuously being remodelled in a process by which osteoclasts resorb bone tissue and osteoblasts produce new bone matrix that is subsequently mineralised. Bone loss occurs when the balance shifts toward excess resorption [4]. In RA, the main reason for bone loss is increased bone resorption, secondary to cytokine-activated osteoclasts, although data on bone formation are conflicting [1].

It has been suggested that treatment which reduces inflammatory activity in RA may prevent bone loss [5]. Therefore, short-term treatment with infliximab has beneficial effects on markers of bone metabolism in patients with active RA [6].

In recent years, glucocorticoid treatment in low doses has been re-evaluated because of its ability to reduce radiographic damage in early RA [7-9]. The side effects of glucocorticoids in RA are still debated, especially the effects on skeletal tissue [10-13].

Glucocorticoid treatment suppresses bone formation by its effects on the osteoblasts. It also changes the micro-architecture and hence the quality of bone, which increases the fracture risk independent of bone mineral density (BMD) [14]. However, in low doses (≤ 7.5 mg) it has been suggested that this suppressive effect on bone synthesis may be compensated by the ability of glucocorticoids to hamper disease activity and thus the inflammatory mediated increase in bone resorption [15].

Fracture risk and osteoporosis are strongly related to BMD, which is therefore widely estimated by dual-energy X-ray absorptiometry (DXA). As a complement to the static measurement of BMD, analyses of biochemical markers of bone turnover are used, because these are useful in predicting future changes in bone mass [16,17]. Also, high bone turnover, detected by biochemical bone markers, might be related to deleterious bone architecture, not detected by DXA [4]. However, the role of assays of bone turnover markers for detection of the effects of glucocorticoids on bone has not been established.

Several markers have been described to measure bone metabolism but it has been difficult to differ between different mechanisms of bone resorption. The type I collagen telopeptide fragments, C-telopeptide crosslaps of type I collagen (CTX-1) and C-terminal telopeptide of type I collagen (1CTP), are currently considered as the most sensitive markers of bone resorption and are released from bone type I collagen by different enzymatic pathways. CTX-1 is generated by cathepsin K, which is the key osteoclastic enzyme for systemic bone resorption. In contrast, 1CTP is generated by matrix-metalloproteases (MMPs) whose activity plays an important role in collagen degradation associated with RA [18,19].

Insulin-like growth hormone (IGF-1) is considered to be the most important skeletal growth factor and it stimulates synthesis of collagen type I, increases matrix apposition rates and inhibits collagen degradation, effects that are important for bone synthesis and maintenance of bone matrix [20]. Glucocorticoids inhibit IGF-1 activity and it is postulated that a reduction in local IGF-1 synthesis is responsible for the inhibition of bone formation caused by steroids [20]. There are limited data about IGF-1 and its association with BMD in RA [21].

The present study on patients with early RA was performed to investigate: the effects of glucocorticoid treatment on bone remodelling markers in relation to BMD; to find possible predictors of changes in BMD; and to study the association of IGF-1 in relation to bone markers and BMD.

## Materials and methods

### Patients

A total of 150 patients (67% women) of the 250 patients with early RA who participated in the BARFOT (Better Anti-Rheumatic FarmacOTherapy) low-dose prednisolone study (a Swedish multi-centre study designed to investigate clinical and therapeutic aspects of early RA) were included in this study [22] between 1995 and 1999. Details of the study protocol have been previously described [9]. Briefly, these patients had a diagnosis of RA according to the American College of Rheumatology criteria [23], were between 18 and 80 years of age, had a disease duration of less than one year and had active disease defined as a Disease Activity Score of 28 joints (DAS28) of more than 3.0. Exclusion criteria were earlier treatment with glucocorticoids and for patients younger than 65 years a T-score lower than -2.5 SD on DXA or for patients 65 years and older a Z-score of less than -1.0 SD. The patients were randomised to treatment with 7.5 mg prednisolone daily (P-group) or no prednisolone (NoP-group) when they started their first disease-modifying anti-rheumatic drug (DMARD), which was chosen by the treating physicians, in accordance with the recommended treatment strategy in Sweden at the time of the study. According to the protocol all patients should be prescribed 1 g calcium daily. Treatment with non-steroidal anti-inflammatory drugs was permitted and intra-articular steroid treatment was also allowed. The patients were followed prospectively for two years.

To be included in this evaluation study, patients should had DXA measurements and X-rays of their hands and feet performed at baseline and at two years follow-up. The final numbers of patients were 70 patients in the P-group and 80 patients in the NoP-group.

The study was approved by the ethics committee at Karolinska University Hospital (KI 153-95), Lund University Hospital (LU 154-95), Sahlgrenska University Hospital in Gothenburg (Gbg M45–95) and Linköping University Hospital (Li 123-95), and was performed according to the Helsinki declaration. All patients signed informed consents.

### Clinical assessments

Disease activity was assessed with C-reactive protein (CRP) and with DAS28, calculated from the number of swollen and tender joints, the erythrocyte sedimentation rate (ESR) and the patient's global assessment of health measured on a visual analogue scale (1 to 100 mm) [23]. Active disease was defined as DAS28 more than 3.0. Functional status was measured using the Swedish version of the Stanford Health Assessment Questionnaire (HAQ) [24]. The HAQ score ranges from 0 to 3, where a higher score indicates a higher degree of disability. Body mass index (BMI) was calculated by dividing body weight by the square of height in metres (kg/m^2^).

### Dual-energy X-ray absorptiometry

Bone mineral density (BMD) was measured at each centre by DXA with a densitometer (GE-Lunar Progidy, Madison, Massachusetts, USA) at the lumbar spine (L2 to L4) with anterior-posterior view and at the left hip (femoral neck) [25]. BMD was expressed in grams of bone mineral per square centimetre (g/cm^2^), as the number of standard deviations (SD) from the mean of healthy age- and sex-matched people, the Z-score, and as the number of SD from the mean of healthy, young sex-matched people, the T-score. The values were obtained from Lunars combined European/US reference population [26].

### Markers of bone turnover

Serum samples were obtained between 9 am and 3 pm and stored at -70°C until assay. For most patients the samples were taken at the same time of the day. All samples from the individual patient were analysed simultaneously to minimise inter-assay variations. The bone markers were analysed at the study centre for laboratory medicine, Karolinska university laboratory, Stockholm, Sweden.

Procollagen type I N-terminal propeptide (P1NP) was used as a marker of bone formation, and CTX-1 and 1CTP as markers of bone degradation. P1NP was determined by Elecsys 1010/2010 total P1NP serum kit (Roche Diagnostics, Mannheim, Germany), which employs the electrochemiluminescence immunoassay (ECLIA) technique. The measuring range is 5 to 1200 μg/L. The median and the 5th and 95th percentiles in premenopausal women are 27.8, 15.13 and 58.59, respectively. For postmenopausal women the corresponding figures are 37.09, 16.27 and 73.87, respectively. The intra-assay coefficients of variation (CV) is 2.3% and total precision CV 2.9%.

CTX-1 was determined by Elecsys 1010/2010 β-CrossLaps/serum kit (Roche Diagnostics, Mannheim, Germany), which also employs the ECLIA technique. The sensitivity of the assay is 0.01 ng/ml. The mean (SD) and mean +2SD figures are: for premenopausal women 0.299 (0.137) ng/mL and 0.573 ng/mL; for postmenopausal women 0.556 (0.226) ng/mL and 1.008 ng/mL; for men 30 to 50 years 0.300 (0.142) ng/mL and 0.584 ng/mL; men 50 to 70 years 0.304 (0.200) ng/mL and 0.704 ng/mL; and for men older than 70 years 0.394 (0.230) ng/mL and 0.854 ng/mL. The intra-individual CV is 17.9%. The intra-assay CV is 2.4% and total precision CV is 3.1%.

1CTP was determined by Multigamma radioimmunoassay kit (Orion Diagnostica, Espoo, Finland). The sensitivity of the assay is 0.5 μg/L. The reference interval is 1.8 to 5.0 μg/L. The intra- and interassay CV is 5.3% and 4.25%, respectively.

### Cytokine

Interleukin (IL) 6 was analysed at the study centre for laboratory medicine, Karolinska university laboratory, Stockholm, Sweden, and was determined in serum by the commercial kits Quantikine HS human IL-6 immunoassay (R& D Systems, Inc., Minneapolis, MN, US), which is a solid-phase ELISA that employs the quantitative sandwich enzyme immunoassay technique. The sensitivity of the assay is 0.039 pg/ml. The mean and range of IL-6 are 1.77 pg/ml and 0.447 to 9.96 pg/ml. The intra- and interassays CV are 7.4% and 7.8%, respectively.

### Anabolic factors

IGF-1 was analysed at the Centre for Diabetes Research, Department of Molecular Medicine and Surgery, Karolinska Institutet, Stockholm, Sweden. IGF-1 in serum was determined by radioimmunoassay (RIA) after separation of IGFs from its regulating binding proteins (IGFBPs) by acid ethanol extraction and cryoprecipitation. To minimise interference of remaining IGFBPs, des (1–3) IGF-1 was used as radioligand [27]. The intra- and interassay CV are 4% and 11%. As serum levels of IGF-1 are age dependent, decreasing with age, IGF-1 values were also expressed as SD scores calculated from the regression of the values of 247 healthy adult subjects [28].

### Statistical analysis

STATISTICA, release 7 (Stat Soft Scandinavia AB, Tulsa, OK, USA) was used for statistical analysis. Data were presented as mean (confidence interval (CI)) or median (interquartile range) depending on its distribution. Comparison between groups was performed with Mann-Whitney U test because many variables were not normally distributed. When comparing two binary variables Fisher's exact test or chi squared test were performed. Correlation analyses were performed with Spearman Rank Order Correlations. We used a Wilcoxon matched pairs test to compare changes between different time-points. Multivariate analyses were performed with multiple linear regression. Changes in BMD at the lumbar spine with respect to the femoral neck were chosen as dependent variables. We used stepwise forward regression analyses to find the most robust model. Variables known to be important for osteoporosis were included in the final models: age, gender, smoking and menopausal status.

## Results

Demographic and clinical variables at baseline are presented in Table 1. The prednisolone-treated group (P-group) was significantly younger and had fewer postmenopausal women compared with the no prednisolone group (NoP-group). Disease activity, Z-scores and bone remodelling markers were similar at baseline. The median levels of all the bone markers were in the normal range compared with the reference values.

**Table 1:** Baseline characteristics of the 150 patients treated with or without prednisolone

	Prednisolone n = 70	No prednisolone n = 80	p level
Age, years	51.3(48.2 to 54.4)	58.4 (55.6 to 61.2)	**0.001**
Disease duration, months	6.3 (5.5 to 7.1)	5.7 (5.1 to 6.4)	0.27
Women, %	69	65	0.64
Menopause, %	50	69	**0.05**
Menopause age, years*	50(48 to 51)	50(47 to 52)	0.97
Current or previous smoker, %	67	56	0.13
BMI*, kg/m^2^	24.3 (22.5 to 27.0)	25.6 (23.5 to 29.1)	0.07
RF positivity, %	66	55	0.25
Erosive disease, %	53	56	0.68
DAS28	5.2 (4.9 to 5.5)	5.5 (5.3 to 5.7)	0.12
ESR	39(33 to 44)	39(33 to 45)	0.96
CRP*	22(10 to 45)	21(10 to 58)	0.50
IL-6*	9.9(8.0 to 19.4)	11.7(9.2 to 27.6)	0.44
HAQ	1.0 (0.9 to 1.2)	1.0 (0.8 to 1.1)	0.52
Z-score, L2–L4*	0.36 (-0.24 to 0.88)	0.50 (-0.36 to 1.37)	0.29
Z-score neck*	0.01 (-0.56 to 1.00)	-0.02 (-0.52 to 0.69)	0.87
P1NP*, μg/L	38.3 (28.0 to 51.8)	40.8 (28.8 to 57.0)	0.50
CTX-1*, ng/mL	0.26 (0.20 to 0.35)	0.33 (0.19 to 0.42)	0.17
1CTP* μg/L	4.5 (3.5 to 5.7)	4.2 (3.4 to 6.4)	0.62
IGF-1, μg/L*	173 (135 to 202)	210 (185 to 250)	0.34
IGFSD	0.15 (-0.06 to 0.6)	1.0 (0.4 to 1.6)	0.42

Data presented as mean (95% confidence interval) or median (interquartile range).

* depending on its distribution.

BMI = body mass index; CRP = C-reactive protein; 1CTP = C-terminal propeptide of type I collagen; CTX-1 = C-terminal telopeptide of type I collagen; DAS = disease activity score; ESR = erythrocyte sedimentation rate; HAQ = health assessment questionnaire; IGF-1 = insulin-like growth factor 1; IGFSD = IGF-1, standard deviation-scores; IL-6 = interleukin-6; P1NP = Procollagen type I N-terminal propeptide; RF = rheumatoid factor. Bold p values are statistically significant.

### Concomitant treatment

DMARDs were given to all patients at baseline. In the P-group, 49% started with methotrexate and 39% with sulfasalazine. The corresponding percentages for the NoP-group were 44% and 45%, respectively. At the two-year visit, several patients in each group had switched from treatment with SSZ to methotrexate in most cases but also to other or no DMARDs, and 9% in the P-group and 15% in the NoP-group were no longer receiving DMARDs. One patient was treated with a biological agent from year one to two. None of the patients were treated with bisphosphonates before inclusion in the study or during the two years of the study. At baseline 38% of the postmenopausal women were treated with hormone replacement therapy (41% in the P-group and 36% in the NoP-group with no significant difference between the groups).

### Disease activity

At baseline all patients had high disease activity measured by DAS28, ESR, CRP and IL-6 but, as expected, these measures decreased significantly in both the P and NoP groups after three months (p < 0.001 for DAS28, ESR and CRP in both treatment groups; IL-6 not measured) and after 12 months (for the P-group p < 0.001 for DAS28, ESR and CRP and p = 0.015 for IL-6, for the NoP-group p < 0.001 for DAS28, ESR and CRP and p = 0.13 for IL-6. The difference between the treatment groups was in favour of the P-group considering ESR reduction at 3 and 12 months (p = 0.001 and p = 0.019, respectively) and a trend also for IL-6 at 12 months (p = 0.051).

### Bone mineral density

Table 2 presents the results of DXA measurements at baseline and the changes after two years. In the P-group, BMD at the lumbar spine had decreased significantly at two years (-2.8%, p < 0.001), although BMD had remained stable in the femoral neck (-0.75%, p = 0.20). In the NoP-group BMD had decreased significantly both at the lumbar spine (-1.1%, p = 0.034) and femoral neck (-1.9%, p < 0.001). Only the reduction in Z-score in the lumbar spine was significantly different between the treatment groups.

**Table 2:** Dual X-ray absorptiometry measurements at baseline and the changes after two years, separated for the two treatment groups

	P-group			NoP-group			P vs NoP
			
	Baseline	Change baseline to 2 years	p value change	Baseline	Change baseline to 2 years	p value change	p value change
BMD, g/cm^2 ^L2 to L4	1.180 (1.070 to 1.263)	-0.032 (-0.063 to 0.010) = -2.8 (-5.9 to 1.0) %	**<0.001**	1.119 (1.063 to 1.227)	-0.013 (-0.054 to 0.025) = -1.1(-4.8 to 2.0) %	**0.034**	0.08
Z-score L2 to L4	0.36 (-0.24 to 0.88)	-0.30 (-0.60 to -0.01)	**<0.001**	0.50 (-0.36 to 1.37)	-0.10 (-0.51 to 0.24)	**0.029**	**0.043**
T-score L2 to L4	-0.17 (-1.03 to 0.56)	-0.30 (-0.51 to 0.06)	**<0.001**	-0.37 (-1.16 to 0.42)	-0.10 (-0.45 to 0.20)	**0.035**	0.07
BMD, g/cm^2 ^neck	0.963 (0.837 to 1.047)	-0.008 (-0.045 to 0.021) = -0.75 (-4.9 to 2.2) %	0.20	0.903 (0.813 to 0.981)	-0.014 (-0.056 to 0.006) = -1.9 (-6.5 to 0.6) %	**<0.001**	0.16
Z-score neck	0.01 (-0.56 to 1.00)	-0.05 (-0.32 to 0.21)	0.43	-0.02 (-0.52 to 0.69)	-0.10 (-0.46 to 0.11)	**0.007**	0.27
T-score neck	-0.50 (-1.66 to 0.30)	-0.03 (-0.39 to 0.18)	0.24	-0.84 (-1.58- to 0.22)	-0.13 (-0.46 to 0.05)	**<0.001**	0.12

Data is presented as median (interquartile range). Changes between baseline and two years are expressed as absolute difference for Z- scores and T-scores and for bone mineral density (BMD) also in percent. P = prednisolone-treated group, No-P = No prednisolone treated group. Bold p values are statistically significant.

As age and percentage of postmenopausal women differed between the treatment groups, we also performed the statistical analyses with women around menopause excluded (age between 47 and 52 years, 10 women in the P-group, 6 women in the NoP-group). We then found no significant differences between the treatment groups in changes in BMD or Z-score (baseline 24 months), either in the lumbar spine or femoral neck, data not shown.

Looking at the groups with those women around menopausal age excluded, there were significant differences when looking at changes in BMD at the lumbar spine between the women in the P-group and those in the NoP-group (median -3.8% (-5.9 to -0.3%) and median -0.3% (-2.4 to 2.3%), respectively (p = 0.004)). The same was found for Z-scores at the lumbar spine median -0.30 (-0.60 to -0.10) and median 0.00 (-0.29 to 0.29), respectively, p = 0.007). At the femoral neck there was no significant differences in changes in BMD or Z-score for women, with those around menopausal age excluded (p = 0.63 and 0.92, respectively). No difference between the treatment groups was shown for premenopausal women or for men either in the lumbar spine or femoral neck (data not shown).

During the two-year study period, there were five new fractures in the P-group (all vertebral, and also a hip fracture for one patient) and six new fractures in the NoP-group (four vertebral, one fracture of the humerus and one fracture of the ankle joint) with no significant difference between the treatment groups.

### Bone turnover markers

At baseline, the median serum levels of all the bone markers were within the normal reference ranges. However, 11% of the women had elevated P1NP (reference values for men not available), 8% of the patients had elevated CTX-1 and 38% had elevated 1CTP.

**Figure 1:**
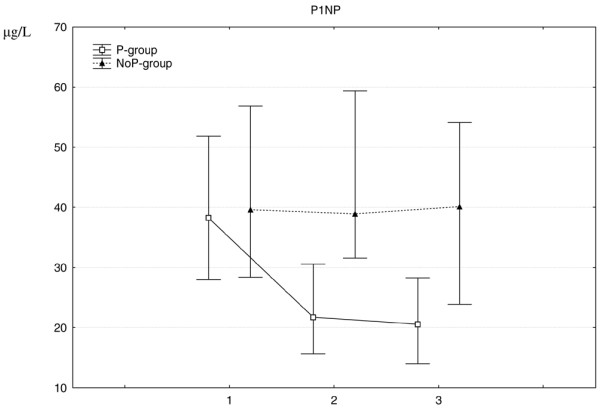
**Change in procollagen type I N-terminal propeptide (P1NP) median values (IQR) at 0, 3 and 12 months for both treatment groups**. P1NP is a marker of bone formation. The difference between the treatment groups was significant both at 3 months (p < 0.001) and 12 months, (p < 0.001). 1 = baseline, 2 = 3 months, 3 = 12 months.

**Figure 2:**
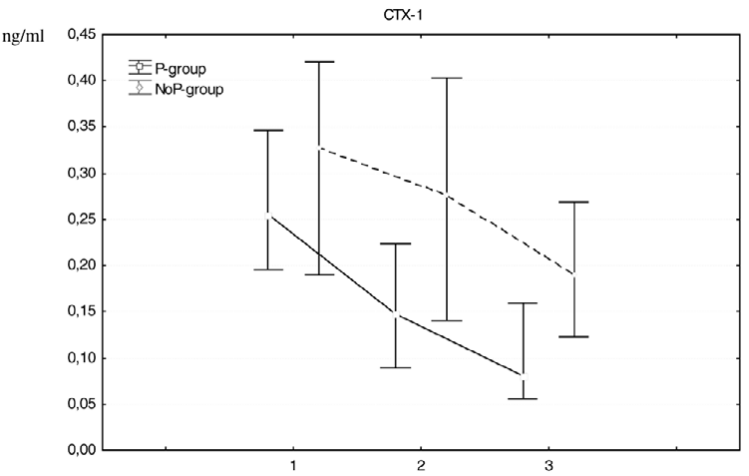
**Change in C-terminal telopeptide cross-laps (CTX-1) median values (IQR) at 0, 3 and 12 months for both treatment groups**. CTX-1 is a marker of bone resorption. The difference between the treatment groups was significant both at 3 months (p = 0.005) and 12 months (p = 0.019). 1 = baseline, 2 = 3 months, 3 = 12 months.

**Figure 3:**
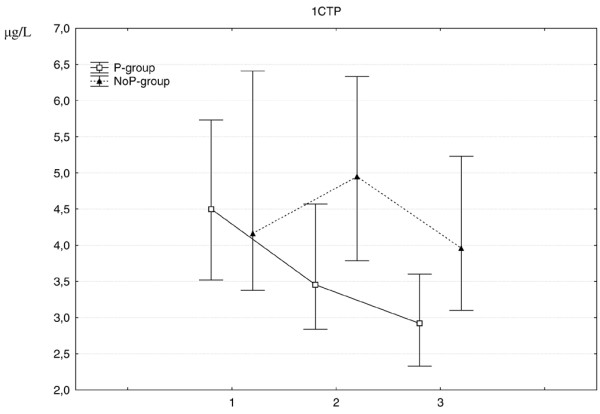
**Change in C-terminal telopeptide of type I collagen (1CTP) median values (IQR) at 0, 3 and 12 months for both treatment group*s***. 1-CTP is a marker of bone resorption. The difference between the two treatment groups was significant, both at 3 months (p < 0.00) and 12 months (p < 0.001). 1 = baseline, 2 = 3 months, 3 = 12 months.

In the P-group, the marker for bone synthesis decreased rapidly during the first three months and more slowly thereafter (Figure 1), whereas the bone resorption markers decreased during the whole 12-month period (Figures 2 and 3).

In the NoP-group, the level of P1NP remained stable for the total period (Figure 1). CTX-1 remained stable for the first three months but decreased thereafter (Figure 2). 1CTP first increased which was then followed by a decrease for the total period (Figure 3).

When comparing the treatment groups, there were significant differences concerning the changes for all the bone markers. The decrease in bone formation occurred only in the P-group and furthermore the decreases in the bone resorption markers were significantly more pronounced and occurred earlier in the P-group (Figures 1, 2 and 3).

### Correlations between bone markers and bone mineral density

At baseline there were no significant correlations between the levels of bone markers and baseline BMD at either skeletal site.

Results of the univariate analyses are shown in Table 3. A decreased bone turnover was associated with less loss of bone, which affected the treatment groups differently. In the P-group, mainly reduced levels of P1NP and 1CTP correlated with changes in BMD at both skeletal sites. In contrast, in the NoP-group, reduced levels of all the bone markers correlated with changes in BMD at the lumbar spine, whereas only reduced 1CTP correlated with changes in BMD at the femoral neck.

**Table 3:** Correlations between bone markers, inflammatory variables and IGFSD at different time-points and changes in bone mineral density during 24 months for the two treatment groups

	Prednisolone group		No prednisolone group	
		
	Δ BMD L2 toL4, 0 to 24 months	Δ BMD neck, 0 to 24 months	Δ BMD L2 to L4, 0 to 24 months	Δ BMD neck, 0 to 24 months
Age	ns	Ns	ns	ns

BMI, baseline	ns	r = 0.26, **p = 0.031**	ns	r = 0.39, **p < 0.001**

P1NP, baseline	ns	Ns	r = -0.36, **p = 0.002**	ns

P1NP, 12 months	r = -0.24, p = 0.057	r = -0.30, **p = 0.018**	r = -0.37, **p = 0.002**	ns

ΔP1NP, 0 to 12 months	ns	ns	ns	ns

CTX-1, baseline	ns	ns	ns	ns

CTX-1, 12 months	ns	ns	r = -0.36, **p = 0.002**	ns

ΔCTX-1, 0 to12 months	ns	r = -0.26, **p = 0.045**	ns	ns

1CTP, baseline	ns	ns	ns	r = -0.27, **p = 0.020**

1CTP, 12 months	ns	r = -0.24, p = 0.059	r = -0.28, **p = 0.017**	r = -0.22, p = 0.072

Δ1CTP, 0 to 12 months	r = -0.29, **p = 0.026**	r = -0.33, **p = 0.010**	ns	Ns

ESR, baseline	ns	ns	ns	r = -0.24, p = 0.031

ESR, 12 months	ns	ns	r = -0.33, **p = 0.004**	r = -0.22, p = 0.053

CRP, baseline	ns	ns	ns	r = -0.28, **p = 0.012**

CRP, 12 months	ns	ns	r = -0.28, **p = 0.015**	r = -0.30, **p = 0.008**

IL-6, baseline	ns	ns	ns	ns

IL-6, 12 months	ns	r = -0.25, **p = 0.037**	ns	ns

IGFSD, baseline	r = 0.36, **p = 0.003**	ns	ns	ns

IGFSD, 12 months	ns	ns	ns	ns

ΔIGFSD, 0 to 12 months	r = -0.31, p = **0.017**	ns	ns	r = -0.25, **p = 0.038**

BMI = body mass index; CRP = C-reactive protein; 1CTP = C-terminal propeptide of type I collagen; CTX = C-terminal telopeptides crosslaps; ESR = erythrocyte sedimentation rate; IGFSD = insulin-like growth factor-1, standard deviation scores; IL-6 = interleukin-6; ns = not significant; P1NP = Procollagen type I N-terminal propeptide. Bold p values are statistically significant.

### Correlations between inflammatory activity and bone mineral density

Results of the univariate analyses are shown in Table 3. Decrease of inflammation correlated in the P-group mainly with less bone loss in the femoral neck, whereas for the NoP-group it was correlated with less bone loss both in the spine and femoral neck.

### Correlations between inflammatory activity and bone markers

Including all patients, clinical and biochemical inflammatory markers correlated positively with bone resorption markers at baseline. The highest correlations were found between 1CTP and CRP (r = 0.48, p < 0.001) and IL-6 (r = 0.34, p < 0.001).

For the P-group reduction of CRP from 0 to 3 months correlated significantly with reduction of CTX-1 during the same time (r = 0.27, p = 0.042), as well as with reduction of 1CTP (r = 0.35, p = 0.007). Reduction in CRP from 0 to 12 months correlated with reduction in 1CTP 0 to 12 months (r = 0.40, p = 0.002), but not significantly with a change in CTX-1 at the same time point.

In the NoP-group reduction of CRP from 0 to 3 months correlated with increase in P1NP from 0 to 3 months (r = -0.39, p = 0.001), as well as with increase in 1CTP (r = -0.36, p = 0.003). Reduction of CRP and IL-6 from 0 to 12 months correlated with reduction in CTX-1 from 0 to 12 months (r = 0.35, p = 0.005 and r = 0.34, p = 0.005, respectively), but not with change in 1CTP.

Thus, reduced inflammation 0 to 3 months was associated with different patterns of the bone markers in the two treatment groups. Reduced inflammation at 0 to 12 months correlated with reduced levels of 1CTP in the P-group and with reduced levels of CTX-1 in the NoP-group.

### Anabolic factors

At baseline, IGF-1 was in the normal range, median (interquartile range) 165 (133 to 198) corresponding to IGFSD of 0.15 (-0.5 to 0.7). At 12 months there was a significant increase in IGF-1 (+43; 18 to 76; p < 0.001) and IGFSD (0.90; 0.30 to 1.50; p < 0.001) in the P-group (corresponding figures for the NoP-group were +6; -17 to 29; p = 0.27; and 0.20; -0.40 to 0.70; p = 0.051, respectively) and the changes at 0 to 12 months in both variables were significantly greater in the P-group than in the NoP-group (p < 0.001 for both comparisons).

### Correlations between IGF-1 and bone mineral density and bone markers

At baseline, including all patients, IGF-1 correlated positively with BMD at the lumbar spine and femoral neck (r = 0.20, p = 0.016 and r = 0.17, p = 0.049, respectively). IGFSD at baseline also correlated positively with baseline BMD at the lumbar spine, but not at the femoral neck (r = 0.20, p = 0.017 and r = 0.06, p = 0.50, respectively).

Baseline levels of IGF-1 also correlated negatively with both CTX-1 and 1CTP (r = -0.21, p = 0.012 and r = -0.38, p < 0.001, respectively). IGF-1 at baseline did not correlate with P1NP. Results of the univariate analyses considering IGFSD and changes in BMD for both the treatment groups are shown in Table 3. Also, in the NoP-group a higher IGF-1 at 12 months correlated with better BMD at the femoral neck after two years (r = 0.31, p = 0.009) and a higher IGFSD at 12 months was associated with better BMD at the lumbar spine at two years (r = 0.26, p = 0.029).

Thus, higher levels of IGF-1 and IGFSD at baseline for all patients and higher levels of IGF-1 and IGFSD at 12 months for the NoP-group were associated with better BMD at both skeletal sites. However, an increase in IGFSD from 0 to 12 months was associated with a loss of bone at the lumbar spine for the P-group and a loss of bone at the femoral neck for the NoP-group. In the P-group, a higher IGF-1 at 12 months was associated with a smaller decrease in 1CTP between 0 to 12 months (r = 0.27, p = 0.042). In the NoP-group, a higher IGF-1 at 12 months was associated with a more pronounced decrease in 1CTP between 0 and 12 months (r = -0.25, p = 0.034).

### Prediction of change in bone mineral density

In multiple-linear regression analysis we used stepwise forward regression with the variables with p < 0.05 from the univariate analysis, as well as variables known to be important for osteoporosis: age, gender, BMI and smoking. Results of the multiple-linear regression analysis are shown in Table 4.

**Table 4:** Multiple regression analysis with change in BMD from 0 to 24 months at the lumbar spine (L2 to L4) and femoral neck as dependent variables. All variables are adjusted for age, gender, BMI and smoking

	Beta	Std.Err of beta	P-level	Adj R^2 ^value
**Δ BMD L2 to L4, 0 to 24 months All patients**				
CRP 12 months	-0.25	0.09	0.006	14 to 16%
P1NP 12 months	-0.26	0.10	0.009	14 to 16%
Glucocorticoid-treatment (1 = yes, 2 = no)	0.21	0.10	0.036	14 to 16%
IGFSD, baseline	0.17	0.09	0.059	14 to 16%
**Δ BMD L2 to L4, 0 to 24 months P-group**				
Gender (1 = woman, 2 = man)	0.31	0.13	0.022	23 to 27%
1CTP, 12 months	-0.25	0.12	0.045	23 to 27%
IGFSD, baseline	0.43	0.12	0.001	23 to 27%
**Δ BMD L2 to L4, 0 to 24 months NoP-group**				
P1NP, 12 months	-0.37	0.11	0.001	22 to 23%
Gender (1 = woman, 2 = man)	-0.29	0.11	0.010	22 to 23%
**Δ BMD femoral neck, 0 to 24 months All patients**				
BMI	0.31	0.08	<0.001	23%
CRP, 12 months	-0.20	0.08	0.017	23%
1CTP, 12 months	-0.26	0.09	0.006	23%
**Δ BMD femoral neck, 0 to 24 months P-group**				
BMI	0.37	0.14	0.010	17 to 20%
1CTP, 12 months	-0.33	0.12	0.012	17 to 20%
**Δ BMD femoral neck, 0 to 24 months NoP-group**				
CRP, 12 months	-0.27	0.11	0.022	20 to 23%
BMI	0.35	0.11	0.003	20 to 23%
1CTP, 12 months	-0.26	0.13	0.050	20 to 23%

BMD = bone mineral density; BMI = body mass index; CRP = C-reactive protein; 1CTP = C-terminal propeptide of type I collagen; IGFSD = insulin-like growth factor-1, standard deviation scores; P1NP = Procollagen type I N-terminal propeptide.

In the lumbar spine higher levels of bone markers and treatment with glucocorticoids were associated with lower BMD. Impact of gender was different in the two treatment groups; in the P-group women lost more bone and men lost more bone in the NoP-group. A higher baseline, IGFSD was associated with higher BMD in the P-group. Adjusting for age, gender, smoking or BMI did not change the correlations. Adding menopausal status to the models strongly affected them resulting in an increased adjusted R^2 ^value of 0.31 for the whole group.

At the femoral neck, higher BMI was associated with higher BMD and a higher level of 1CTP at 12 months was associated with more bone loss for both groups. Treatment with glucocorticoids had no impact on bone loss at the femoral neck, nor did gender in either group. Including age, smoking or menopausal status in the model did not change the correlations.

When looking at gender differences, the most important finding was that in women, glucocorticoid treatment predicted 0 to 24 months change in BMD at lumbar spine (regression coefficient β = 0.55, standard error of β = 0.10, p < 0.001).

## Discussion

In this prospective, open study of early, active RA over two years the patients who were in the original study were randomised to 7.5 mg prednisolone or no prednisolone when they started their treatment with DMARD. This treatment could not prevent bone loss in the spine, in spite of decreased inflammation and decreased bone resorption. In the P-group, bone loss was prevented in the femur compared with the NoP-group where BMD also decreased in the hip.

The decrease in BMD at the lumbar spine was more pronounced in women in the P-group, especially in the postmenopausal women. This difference was evident even when excluding women around the age of menopause, a period with early menopause-associated trabecular BMD loss. In premenopausal women and men, there was no difference in lumbar spine bone loss between the treatment groups. It thus seems that menopausal status was a risk factor for bone loss in the spine in combination with treatment with glucocorticoids. Previous results concerning the effects of menopausal status in glucocorticoid-treated RA patients were conflicting [29,30].

The fact that bone loss in the P-group was only evident in the spine and not in the femur might partly depend on the fact that trabecular bone, which has a higher bone turnover than cortical bone [31], is more abundant in the spine than in the femoral neck [32]. This makes the spine more vulnerable when the balance in bone remodelling changes towards decreased bone formation, a known effect of glucocorticoids [33] also shown here. Furthermore, postmenopausal osteoporosis also mainly affects the trabecular bone [34], and the combined effect of postmenopausal status and glucocorticoid treatment may increase the vulnerability.

Median levels of the bone markers were not elevated (compared with the reference values) in spite of active disease. This differs from some earlier reports of increased levels in RA patients with active disease [35,36] but not from all [37]. Here 1CTP was elevated in 38% of the patients at baseline, while CTX-1 was only elevated in 8%, percentages close to those reported earlier [37]. 1CTP mirrors the inflammatory mediated local bone resorption in the arthritic joints, suggested to be prominent early in disease, more than the systemic bone resorption such as osteoporosis [38,39] reflected by CTX-1 elevation. The changes in bone resorption markers over time correlated with reduced inflammation, which has also been showed by other groups [5,6,19].

The marker of bone formation, P1NP, decreased in the P-group but remained stable in the NoP-group, which is in accordance with the known effects of glucocorticoids on bone. Glucocorticoids thus inhibit bone formation by modifying osteoblastic cell differentiation, number and function [33]. In a study of healthy volunteers, treatment with 10 mg prednisolone resulted in a decreased levels of bone formation markers and, to a smaller degree, of 1CTP [40].

The bone resorption markers decreased in both groups but with different patterns. Thus CTX-1 decreased rapidly within the first three months, which was more pronounced in the P-group. This quick response is similar to that achieved by infliximab treatment [19] but in contrast to that treatment the values did not return to pre-treatment levels after 12 months. The 1CTP level decreased more slowly and in the NoP-group was only significant at 12 months, also in line with infliximab treatment [19]. The different patterns suggest that CTX-1 and 1CTP reflect different resorption processes. 1CTP is preferentially localised in local joints [37] and tumour necrosis factor blockade inhibits activity of the 1CTP-generating MMPs [41]. The greater reduction of 1CTP found here in the P-group compared with the NoP-group indicates a more pronounced inhibition of MMPs induced by prednisolone. This inhibition might not only explain the earlier reported ability of prednisolone to reduce radiographic damage in the joints of hands and feet in the RA patients [9], but also the finding that bone resorption was only inhibited in the P-group in the femur, a juxta-articular localisation.

In the P-group, CTX-1 seemed to decrease more than P1NP, similar to what was observed with infliximab treatment [19]. This means that the reduction of P1NP levels in the P-group was balanced by the decrease in CTX-1. In contrast, 1CTP did not seem to decrease as much as P1NP indicating that the reduced bone formation in the P-group was not balanced by the reduced 1CTP.

At baseline, only levels of P1NP correlated with changes in BMD of the lumbar spine two years later. Adequate thresholds for bone markers are lacking and the current data do not indicate that bone markers at baseline can predict the rate of bone loss in an individual with sufficient accuracy to be used in clinical practice [16]. That suppression of bone resorption is important was, however, shown as levels of bone markers at 12 months, or the change from 0 to 12 months, predicted bone loss. This was shown for both treatment groups at both skeletal sites.

Most clinical studies have shown a positive correlation between IGF-1 and BMD [42]. Like others, we also found a positive correlation between IGF-1 and BMD at both skeletal sites at baseline. IGF-1 increased significantly only in the P-group. This is in line with other groups who have also found an association between treatment with glucocorticoids and increased levels of IGF-1 [43-46]. In patients with Cushing's syndrome, IGF-1 has also been found to be elevated [47]. A possible explanation for this unexpected increase in IGF-1 could be that glucocorticoids inhibit the production of IGF-1 in the muscle, followed by a decrease in negative feedback regulation of liver IGF-1, which would increase serum levels of IGF-1 [46]. In correlation analyses, there were a negative correlation between changes in IGF-1 and changes in BMD at the lumbar spine for the P-group and at the femoral neck for the NoP-group. Treatment with glucocorticoids can be associated with IGF-1 resistance [43,44,46], similar to insulin resistance, which may explain the negative correlation in the P-group.

Canalis and Avioli have postulated that reduced IGF-1 synthesis may be responsible for the inhibition of bone formation caused by steroids [48]. In the present study we did not find any significant correlation between IGF-1 and markers of bone formation, although such a correlation was found between IGF-1 and the bone resorption markers. The lack of significant correlation between IGF-1 and the bone formation marker may be because the effects of IGF-1 on bone occur in a paracrine or autocrine manner [20]. The fact that in the NoP-group we found a negative correlation between changes in IGF-1 and changes in BMD at the femoral neck could be due to the fact that IGF-1 resistance may be secondary to inflammatory activity, just like the peripheral insulin resistance. Research over the past decade indicates the ability of pro-inflammatory cytokines to induce a state of IGF-1 resistance [49].

The primary limitation of this study is that not all patients in the randomised study of low-dose prednisolone were included in this mechanistic study. The reason for this was that only patients with DXA and radiographs at both baseline and two years were eligible. This implies that although the study was randomised the number of patients excluded was different between treatment arms, which must be considered when interpreting the results. Furthermore the randomised study was designed to study radiological progression. When designing studies on bone density great efforts must be devoted to comparisons of groups with mixed gender, age and different menopausal status. However, being aware of these shortcomings, we tried to adjust for these differences in the analyses. Further, the blood for bone turnover markers was not obtained in the fasting state, which may have affected some of the results. Blood samples were usually drawn at the same time for each individual patient. Another limitation of this study is that no placebo was used.

## Conclusion

The data presented indicate that glucocorticoids counteract the negative impact of rheumatoid inflammation on bone tissue. In the hip with its bone tissue close to a large joint, BMD was preserved in the P-group. However, in the spine, a skeletal site not directly affected by the synovial inflammatory process in RA, glucocorticoid treatment was associated with a decrease in BMD. In the light of these data we suggest that glucocorticoids may protect against the negative effects on bone primarily caused by the rheumatoid disease, a suggestion supported by the rapid decrease in 1CTP in the P-group.

On the other hand, the systemic inflammatory consequences on bone could not be prevented because of suppression of bone synthesis. Bone loss in the spine was more pronounced in postmenopausal women treated with glucocorticoids. Bone protection for this group of patients should always be considered, especially if glucocorticoids are given.

## Abbreviations

BARFOT: Better Anti-Rheumatic FarmacOTherapy; BMI: body mass index; BMD: bone mineral density; CI: confidence interval; CRP: C-reactive protein; 1CTP: C-terminal telopeptide of type I collagen; CTX-1: C-telopeptide crosslaps of type I collagen; DAS28: disease activity score of 28 joints; DMARD: disease-modifying anti-rheumatic drug; DXA: dual-energy X-ray absorptiometry; ESR: erythrocyte sedimentation rate; HAQ: health Assessment Questionnaire; IGF-1: insulin-like growth factor-1; IGFSD: IGF standard deviations; IL: interleukin; IQR: inter quartile range; MMPs: matrix- metalloproteases; NoP-group: no prednisolone treated group; P-group: prednisolone treated group; P1NP: procollagen type I N-terminal propeptide; RA: rheumatoid arthritis.

## Competing interests

The authors declare that they have no competing interests.

## Authors' contributions

ILE performed the searches, data analyses, statistical analyses and prepared the manuscript. BT was involved in the statistical analyses and helped to draft the manuscript. BS and IH were involved with the original concept, planning the study and statistical analyses, as well as with preparing the manuscript. KB has been involved with analyses and interpretation of the IGF-1 data. All authors read and approved the final manuscript.

## References

[B1] Deodhar AA, Woolf AD (1996). Bone mass measurement and bone metabolism in rheumatoid arthritis: a review. Br J Rheumatol.

[B2] Hooyman JR, Melton LJ, Nelson AM, O'Fallon WM, Riggs BL (1984). Fractures after rheumatoid arthritis. A population-based study. Arthritis Rheum.

[B3] Spector TD, Hall GM, McCloskey EV, Kanis JA (1993). Risk of vertebral fracture in women with rheumatoid arthritis. BMJ.

[B4] Bonnick SL, Shulman L (2006). Monitoring osteoporosis therapy: bone mineral density, bone turnover markers, or both?. Am J Med.

[B5] Dolan AL, Moniz C, Abraha H, Pitt P (2002). Does active treatment of rheumatoid arthritis limit disease-associated bone loss?. Rheumatology (Oxford).

[B6] Vis M, Wolbink GJ, Lodder MC, Kostense PJ, Stadt RJ van de, de Koning MH, Dijkmans BA, Lems WF (2003). Early changes in bone metabolism in rheumatoid arthritis patients treated with infliximab. Arthritis Rheum.

[B7] Kirwan JR (1995). The effect of glucocorticoids on joint destruction in rheumatoid arthritis. The Arthritis and Rheumatism Council Low-Dose Glucocorticoid Study Group. N Engl J Med.

[B8] van Everdingen AA, Jacobs JW, Siewertsz Van Reesema DR, Bijlsma JW (2002). Low-dose prednisone therapy for patients with early active rheumatoid arthritis: clinical efficacy, disease-modifying properties, and side effects: a randomized, double-blind, placebo-controlled clinical trial. Ann Intern Med.

[B9] Svensson B, Boonen A, Albertsson K, Heijde D van der, Keller C, Hafstrom I (2005). Low-dose prednisolone in addition to the initial disease-modifying antirheumatic drug in patients with early active rheumatoid arthritis reduces joint destruction and increases the remission rate: a two-year randomized trial. Arthritis Rheum.

[B10] Sambrook PN, Cohen ML, Eisman JA, Pocock NA, Champion GD, Yeates MG (1989). Effects of low dose corticosteroids on bone mass in rheumatoid arthritis: a longitudinal study. Ann Rheum Dis.

[B11] Laan RF, van Riel PL, Putte LB van de, van Erning LJ, van't Hof MA, Lemmens JA (1993). Low-dose prednisone induces rapid reversible axial bone loss in patients with rheumatoid arthritis. A randomized, controlled study. Ann Intern Med.

[B12] Verhoeven AC, Boers M (1997). Limited bone loss due to corticosteroids; a systematic review of prospective studies in rheumatoid arthritis and other diseases. J Rheumatol.

[B13] Strand V, Simon LS (2003). Low dose glucocorticoids in early rheumatoid arthritis. Clin Exp Rheumatol.

[B14] Mazziotti G, Angeli A, Bilezikian JP, Canalis E, Giustina A (2006). Glucocorticoid-induced osteoporosis: an update. Trends Endocrinol Metab.

[B15] Tengstrand B, Larsson E, Klareskog L, Hafstrom I (2007). Randomized withdrawal of long-term prednisolone treatment in rheumatoid arthritis: effects on inflammation and bone mineral density. Scand J Rheumatol.

[B16] Delmas PD, Eastell R, Garnero P, Seibel MJ, Stepan J (2000). The use of biochemical markers of bone turnover in osteoporosis. Committee of Scientific Advisors of the International Osteoporosis Foundation. Osteoporos Int.

[B17] Nishizawa Y, Nakamura T, Ohata H, Kushida K, Gorai I, Shiraki M, Fukunaga M, Hosoi T, Miki T, Nakatsuka K, Miura M (2001). Guidelines on the use of biochemical markers of bone turnover in osteoporosis (2001). J Bone Miner Metab.

[B18] Garnero P, Ferreras M, Karsdal MA, Nicamhlaoibh R, Risteli J, Borel O, Qvist P, Delmas PD, Foged NT, Delaisse JM (2003). The type I collagen fragments ICTP and CTX reveal distinct enzymatic pathways of bone collagen degradation. J Bone Miner Res.

[B19] Chopin F, Garnero P, le Henanff A, Debiais F, Daragon A, Roux C, Sany J, Wendling D, Zarnitsky C, Ravaud P, Thomas T (2008). Long term effects of Infliximab on bone and cartilage turnover markers in patients with rheumatoid arthritis. Ann Rheum Dis.

[B20] Delany AM, Pash JM, Canalis E (1994). Cellular and clinical perspectives on skeletal insulin-like growth factor I. J Cell Biochem.

[B21] Toussirot E, Nguyen NU, Dumoulin G, Aubin F, Cedoz JP, Wendling D (2005). Relationship between growth hormone-IGF-I-IGFBP-3 axis and serum leptin levels with bone mass and body composition in patients with rheumatoid arthritis. Rheumatology (Oxford).

[B22] Svensson B, Schaufelberger C, Teleman A, Theander J (2000). Remission and response to early treatment of RA assessed by the Disease Activity Score. BARFOT study group. Better Anti-rheumatic Farmacotherapy. Rheumatology (Oxford).

[B23] Arnett FC, Edworthy SM, Bloch DA, McShane DJ, Fries JF, Cooper NS, Healey LA, Kaplan SR, Liang MH, Luthra HS (1988). The American Rheumatism Association 1987 revised criteria for the classification of rheumatoid arthritis. Arthritis Rheum.

[B24] Ekdahl C, Eberhardt K, Andersson SI, Svensson B (1988). Assessing disability in patients with rheumatoid arthritis. Use of a Swedish version of the Stanford Health Assessment Questionnaire. Scand J Rheumatol.

[B25] Lewiecki EM, Baim S, Binkley N, Bilezikian JP, Kendler DL, Hans DB, Silverman S (2008). Report of the International Society for Clinical Densitometry 2007 Adult Position Development Conference and Official Positions. South Med J.

[B26] Lunar (1998). Operator manual, Expert-XL software version 1.7. Lunar Corporation.

[B27] Bang P, Eriksson U, Sara V, Wivall IL, Hall K (1991). Comparison of acid ethanol extraction and acid gel filtration prior to IGF-I and IGF-II radioimmunoassays: improvement of determinations in acid ethanol extracts by the use of truncated IGF-I as radioligand. Acta Endocrinol (Copenh).

[B28] Hilding A, Brismar K, Degerblad M, Thoren M, Hall K (1995). Altered relation between circulating levels of insulin-like growth factor-binding protein-1 and insulin in growth hormone-deficient patients and insulin-dependent diabetic patients compared to that in healthy subjects. J Clin Endocrinol Metab.

[B29] Als OS, Gotfredsen A, Christiansen C (1985). The effect of glucocorticoids on bone mass in rheumatoid arthritis patients. Influence of menopausal state. Arthritis Rheum.

[B30] Butler RC, Davie MW, Worsfold M, Sharp CA (1991). Bone mineral content in patients with rheumatoid arthritis: relationship to low-dose steroid therapy. Br J Rheumatol.

[B31] Delaney MLM, Ruddy S, Harris E, Sledge C (2001). Metabolic Bone Disease. Kelley's Textbook of Rheumatology.

[B32] van Staa TP, Leufkens HG, Cooper C (2002). The epidemiology of corticosteroid-induced osteoporosis: a meta-analysis. Osteoporos Int.

[B33] Canalis E (2003). Mechanisms of glucocorticoid-induced osteoporosis. Curr Opin Rheumatol.

[B34] Khosla S, Melton LJ, Riggs BL (1999). Osteoporosis: gender differences and similarities. Lupus.

[B35] Gough AK, Peel NF, Eastell R, Holder RL, Lilley J, Emery P (1994). Excretion of pyridinium crosslinks correlates with disease activity and appendicular bone loss in early rheumatoid arthritis. Ann Rheum Dis.

[B36] Lems WF, Gerrits MI, Jacobs JW, van Vugt RM, van Rijn HJ, Bijlsma JW (1996). Changes in (markers of) bone metabolism during high dose corticosteroid pulse treatment in patients with rheumatoid arthritis. Ann Rheum Dis.

[B37] Sassi ML, Aman S, Hakala M, Luukkainen R, Risteli J (2003). Assay for cross-linked carboxyterminal telopeptide of type I collagen (ICTP) unlike CrossLaps assay reflects increased pathological degradation of type I collagen in rheumatoid arthritis. Clin Chem Lab Med.

[B38] Kotaniemi A, Isomaki H, Hakala M, Risteli L, Risteli J (1994). Increased type I collagen degradation in early rheumatoid arthritis. J Rheumatol.

[B39] Paimela L, Leirisalo-Repo M, Risteli L, Hakala M, Helve T, Risteli J (1994). Type I collagen degradation product in serum of patients with early rheumatoid arthritis: relationship to disease activity and radiological progression in a 3-year follow-up. Br J Rheumatol.

[B40] Lems WF, Van Veen GJ, Gerrits MI, Jacobs JW, Houben HH, Van Rijn HJ, Bijlsma JW (1998). Effect of low-dose prednisone (with calcium and calcitriol supplementation) on calcium and bone metabolism in healthy volunteers. Br J Rheumatol.

[B41] Catrina AI, Lampa J, Ernestam S, af Klint E, Bratt J, Klareskog L, Ulfgren AK (2002). Anti-tumour necrosis factor (TNF)-alpha therapy (etanercept) down-regulates serum matrix metalloproteinase (MMP)-3 and MMP-1 in rheumatoid arthritis. Rheumatology (Oxford).

[B42] Jassal SK, von Muhlen D, Barrett-Connor E, Rosen CJ (2005). Serum insulin-like growth factor binding protein-1 levels and bone mineral density in older adults: the Rancho Bernardo Study. Osteoporos Int.

[B43] Dong F, Ren J (2003). Insulin-like growth factors (IGFs) and IGF-binding proteins in nephrotic syndrome children on glucocorticoid. Pharmacol Res.

[B44] Zhou X, Loke KY, Pillai CC, How HK, Yap HK, Lee KO (2001). IGFs and IGF-binding proteins in short children with steroid-dependent nephrotic syndrome on chronic glucocorticoids: changes with 1 year exogenous GH. Eur J Endocrinol.

[B45] Prummel MF, Wiersinga WM, Oosting H, Endert E (1996). The effect of long-term prednisone treatment on growth hormone and insulin-like growth factor-1. J Endocrinol Invest.

[B46] Sarzi-Puttini P, Atzeni F, Scholmerich J, Cutolo M, Straub RH (2006). Anti-TNF antibody treatment improves glucocorticoid induced insulin-like growth factor 1 (IGF1) resistance without influencing myoglobin and IGF1 binding proteins 1 and 3. Ann Rheum Dis.

[B47] Bang P, Degerblad M, Thoren M, Schwander J, Blum W, Hall K (1993). Insulin-like growth factor (IGF) I and II and IGF binding protein (IGFBP) 1, 2 and 3 in serum from patients with Cushing's syndrome. Acta Endocrinol (Copenh).

[B48] Canalis E, Avioli L (1992). Effects of deflazacort on aspects of bone formation in cultures of intact calvariae and osteoblast-enriched cells. J Bone Miner Res.

[B49] O'Connor JC, McCusker RH, Strle K, Johnson RW, Dantzer R, Kelley KW (2008). Regulation of IGF-I function by proinflammatory cytokines: At the interface of immunology and endocrinology. Cell Immunol.

